# Validation of Oximetry for Diagnosing Obstructive Sleep Apnea in a Clinical Setting

**DOI:** 10.3390/clockssleep2030027

**Published:** 2020-08-29

**Authors:** Kazuki Ito, Masahiro Uetsu, Hiroshi Kadotani

**Affiliations:** 1 Department of Sleep and Behavioral Sciences, Shiga University of Medical Science, Seta Tsukinowa-cho, Otsu, Shiga 520-2192, Japan; momo3@belle.shiga-med.ac.jp; 2 Department of Anesthesiology, Shiga University of Medical Science, Seta Tsukinowa-cho, Otsu, Shiga 520-2192, Japan; 3 Sleep Outpatient Unit for Sleep Apnea Syndrome, Nagahama City Hospital, 313 Ohinui-cho, Nagahama, Shiga 526-0043, Japan; muetsu@nagahama-hp.jp

**Keywords:** epidemiology, obstructive sleep apnea, oximetry, polysomnography, correlation, ROC curve, Bland-Altman plot, home sleep apnea testing

## Abstract

A large epidemiological study using oximetry to analyze obstructive sleep apnea (OSA) and metabolic comorbidities was performed in Japan; however, reliability and validity of oximetry in the Japanese population remains poorly understood. In this study, oximetry data from the epidemiological study were compared with data from clinically performed polysomnography (PSG) and out-of-center sleep testing (OCST) in epidemiological study participants who later attended our outpatient units. The oxygen desaturation index (ODI) from oximetry showed a moderate positive relationship (correlation coefficient *r* = 0.561, *p* < 0.001) with apnea/hypopnea data from PSG/OCST. The area under the receiver operating characteristic curve showed moderate accuracy of this method in the detection of moderate-to-severe or severe OSA. However, the optimal ODI thresholds to detect moderate-to-severe OSA and severe OSA were the same (ODI > 20.1). Oximetry may be a useful tool for screening moderate-to-severe or severe sleep apnea. However, it may be difficult to set an appropriate threshold to distinguish between moderate and severe sleep apnea by oximetry alone.

## 1. Introduction

Obstructive sleep apnea (OSA) is a common sleep disorder [1,2]; its prevalence is estimated to be as high as 14% in Japan [2]. OSA is an independent risk factor for a variety of adverse metabolic disease states, including hypertension, type 2 diabetes, dyslipidemia, and metabolic syndrome [3,4,5,6,7,8].

Overnight polysomnography (PSG), which is analyzed by a specialized technician, is the gold standard for OSA diagnosis [9]. However, PSG is time- and labor-intensive, and, consequently, waiting times can be substantial [10]. To increase accessibility to diagnostic resources for early detection, the unattended recording of some physiological signals at home has been encouraged [9,11]. Oximetry, one of the simplest methods to obtain physiological signals, has the potential to be a cheaper, more accessible alternative diagnostic tool to screen for OSA, especially in large-scale studies. Many studies using oximetry have investigated the accuracy of the oxygen desaturation index (ODI) as a metric for OSA diagnosis; however, variability has been reported in the specificity (≈40–100%) and sensitivity (≈30–100%) of ODI measurements in oximetry [12,13]. These variations may come, in part, from different study designs. Some studies have compared oximetry data obtained as a part of PSG recordings to apnea events analyzed from the same PSG recordings [14,15,16,17,18,19]. Others have compared oximetry data with data from simultaneous PSG [20,21,22]. These methods may be suitable for developing and comparing algorithms or devices; however, they are not informative enough for clinical settings. In Japan, oximetry is performed during regular annual medical examinations for screening of OSA in clinical settings. After the screening, patients with suspected OSA visit hospitals or clinics with ODI reports and undergo out-of-center sleep testing (OCST) or PSG for diagnosis.

Few studies have compared PSG and oximetry data from different nights [23,24]. Golpe et al. [23] performed a retrospective analysis of 127 patients suspected of having sleep apnea in Spain, in which both home oximetry and PSG were performed. This study compared PSG and oximetry data collected 12.8 ± 10.1 months apart and found that the correlations between the apnea–hypopnea index (AHI) and desaturation indices were not high, with an *r* = 0.58 for ODI at 3% (3%ODI), *r* = 0.60 for desaturations for 4%, and *r* = 0.50 for time spent at saturations below 90%. That study also found that oximetry had a high specificity (0.97) for confirming sleep apnea in patients with high pre-test probabilities of OSA; however, its sensitivity (0.29) was not adequate. Chu et al. [24] performed another prospective, cross-sectional study among 107 hemodialysis patients in Australia, in which PSG was performed only when oximetry presented abnormal results. Ninety-three patients completed nocturnal oximetry, with 65 having abnormal results (3%ODI ≥ 5). Thirty-six patients underwent both oximetry and PSG, with both evaluations being performed on the night after a dialysis session in order to minimize the influence of fluid overload on respiratory function. The time period between oximetry and PSG performance was not presented in the study; however, the average wait time for a non-urgent in-laboratory PSG in Australia is ≤ 68 weeks. The 3%ODI measured from home oximetry and the AHI measured with in-lab PSG were positively correlated (*r* = 0.62, *p* = 0.0001).

Matsumoto et al. [25] published a large epidemiological study (the Nagahama Study) in Nagahama, Japan, in which 7713 participants underwent pulse oximetry in their homes to screen for sleep apnea. In this study, home fingertip oximetry worn on the nondominant wrist was performed with wrist actigraphy. Using actigraphy, OSA was assessed by measuring 3%ODI corrected for sleep duration. Prevalence of moderate (actigraphy-modified 3%ODI: 15–30) and severe OSA (actigraphy-modified 3%ODI ≥ 30) were 10.1% and 2.0%, respectively. This study also found that comorbidities like hypertension, diabetes, and metabolic syndrome were independently associated with moderate-to-severe OSA. As a preliminary study, Matsumoto et al. [25] compared simultaneous in-lab PSG with oximetry and actigraphy among 32 participants. Actigraphy-modified 3%ODI (*r* = 0.99, *p* < 0.001) was more comparable to the AHI recorded from PSG than the non-modified 3%ODI (*r* = 0.92, *p* < 0.001).

In this study, we aimed to compare oximetric data obtained in the Nagahama Study with sleep apnea-related diagnostic data from our clinical setting. Since our clinical unit is the only one in Nagahama that can perform in-lab PSG, Nagahama study participants with suspected sleep apnea were encouraged to consult at our unit. In this study, we compared OSA diagnostic data obtained in our clinical setting to the oximetry data obtained in the Nagahama Study. Since oximetry is much less invasive than monitoring systems currently in use, it is important to determine whether this home-based test can reliably screen for and detect OSA. Importantly, our study sought to evaluate the usefulness of previously performed oximetry data in clinical settings.

## 2. Results

**Figure 1:**
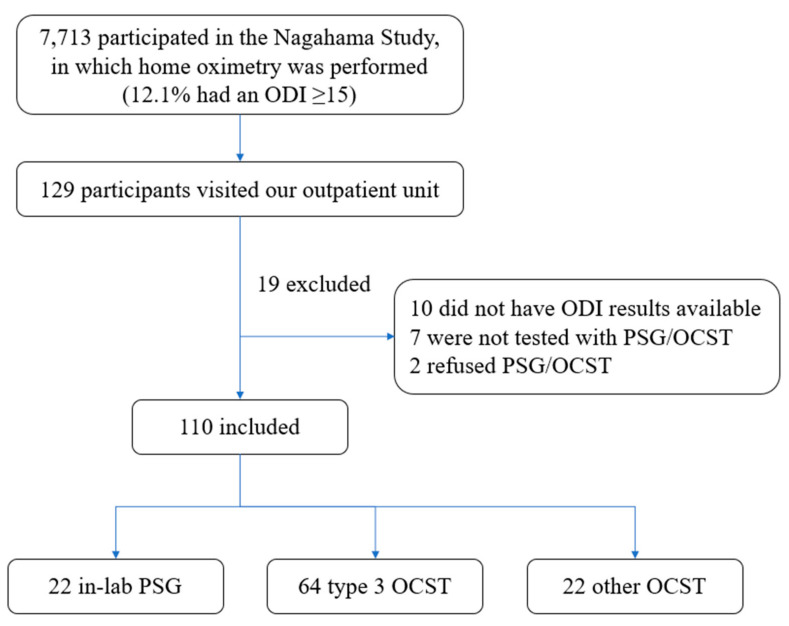
Flow diagram of the participants. ODI: oxygen desaturation index; PSG: polysomnography; OCST: out-of-center sleep testing.

ODI data were available for 119 of the 129 participants in the Nagahama Study, and these were included in our study (Figure 1). The characteristics of participants that were either included or excluded from the study were similar (Table 1).

**Figure 2:**
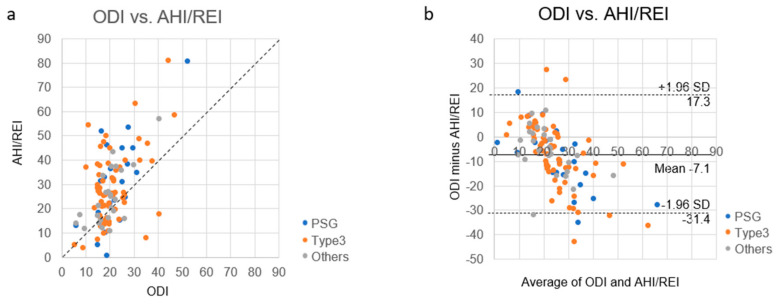
Oxygen desaturation index (ODI) obtained in the Nagahama Study versus apnea-hypopnea index (AHI), analyzed by polysomnography (PSG), and respiratory event index (REI), analyzed by out-of-center sleep testing (OCST) obtained in the clinical setting. (**a**) A scatter plot and (**b**) a Bland–Altman plot were generated to compare ODI and AHI/REI. Blue dots represent AHI from polysomnography (PSG), orange dots represent REI from type 3 OCST (Type 3), and gray dots represent REI from 2–3 channel OCST (Others).

**Figure 3:**
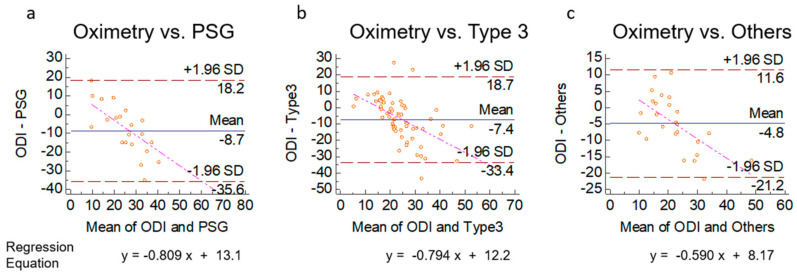
Bland–Altman plots and their regression equations to compare the oxygen desaturation index (ODI) vs. respiratory event index (REI) analyzed with out-of-center sleep testing (OCST) and apnea-hypopnea index (AHI) analyzed with polysomnography (PSG). ODI from oximetry (Oximetry) was compared with (**a**) AHI from PSG, (**b**) REI from type 3 OCST (Type 3), and (**c**) REI from 2–3 channel OCST (Others).

Evaluation of the relationship between ODI and AHI/REI revealed a moderate positive relationship (correlation coefficient *r* = 0.561, *p* < 0.001) (Figure 2a). The Bland–Altman plot suggested that oximetry had systematic errors when compared with PSG or OCST (Figure 2b). Slopes of the regression equations for Bland–Altman plots of PSG, type 3, and “others” versus ODI were −0.809 (95% CI: −1.150 to −0.469), −0.794 (95% CI: −1.069 to −0.519), and −0.590 (95% CI: −0.922 to −0.259), respectively (Figure 3a–c).

**Figure 4:**
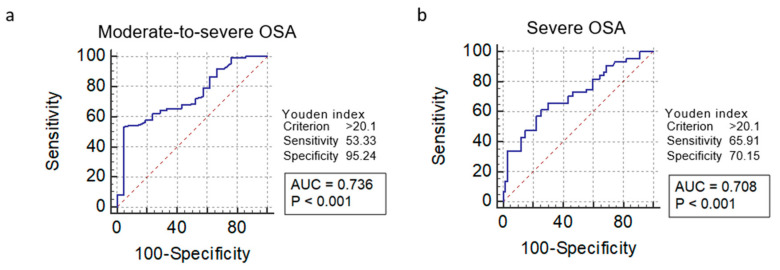
Comparison of moderate-to-severe and severe obstructive sleep apnea (OSA) using the area under the receiver operating characteristics curve (AUC) and Youden index for the oxygen desaturation index. Receiver operating characteristic (ROC) curves were constructed to compare the diagnostic performance of ODI to detect moderate-to-severe (AHI/REI ≥ 15) (**a**) or severe (AHI/REI ≥ 30) OSA (**b**).

The area under the ROC curve (AUC) serves as an overall measure of a diagnostic test’s accuracy. The AUC values suggested that ODI offered a moderate level of accuracy to detect both moderate-to-severe (0.736) and severe (0.708) OSA (Figure 4a,b). The Youden index was used to calculate the optimal ODI thresholds to detect moderate-to-severe OSA (ODI > 20.1) and severe OSA (ODI > 20.1).

According to the previously obtained oximetry data from the epidemiological study, 8.2%, 80.0%, and 11.8% of patients were diagnosed with mild, moderate, or severe OSA (Table 2), respectively. According to PSG/OCST data obtained in our clinical setting, 16.4%, 40.9%, and 40.0% of the same patients were clinically diagnosed with mild, moderate, or severe OSA (Weighted Kappa = 0.238), respectively.

**Figure 5:**
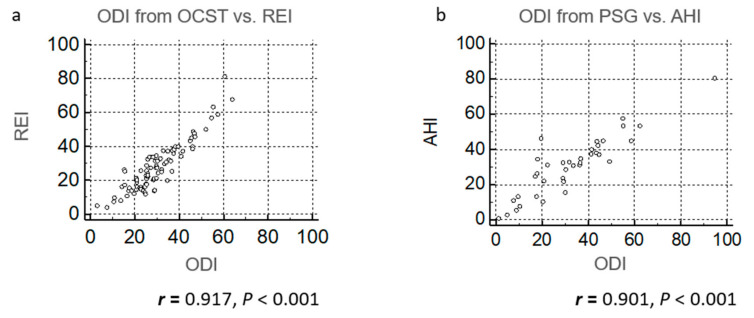
Scatter plots of oxygen desaturation index (ODI) vs. respiratory event index (REI) analyzed with out-of-center sleep testing (OCST) and apnea-hypopnea index (AHI) analyzed with polysomnography (PSG) from the same recording. Oximetry was performed as a part of PSG or OCST. (**a**) ODI from OCST was compared with REI and (**b**) ODI from PSG was compared with AHI.

**Figure 6:**
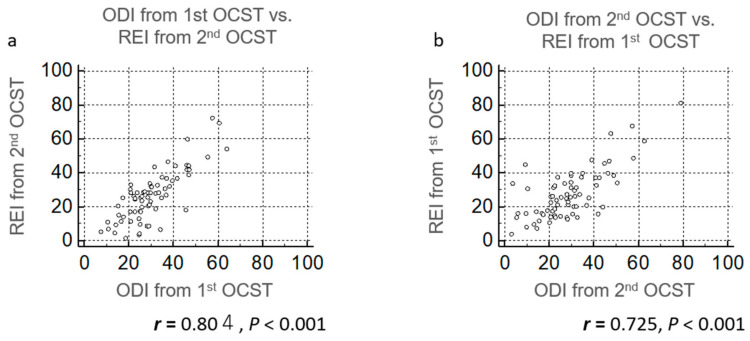
Scatter plots of oxygen desaturation index (ODI) vs. respiratory event index (REI) analyzed with out-of-center sleep testing (OCST) from different recordings. Two nights of OCST were performed. (**a**) ODI from the first night was compared with the REI from the second night and (**b**) ODI from the second night was compared with REI from the first night.

When we compared REI/AHI and ODI derived from the same recordings (ODI from OCST with REI and ODI from PSG with AHI), correlation coefficients were 0.917 and 0.901, respectively (Figure 5a,b). When ODI from the first night of OCST was compared with REI from the second night, the correlation coefficient *r* was 0.804 (*p* < 0.001) (Figure 6a). When ODI from the second night with OCST was compared with REI from the first night, the correlation coefficient *r* was 0.725 (*p* < 0.001) (Figure 6b). Therefore, correlations between REI/AHI and ODI were best when ODI and AHI/REI were analyzed from the same recordings, followed by the same device on different days, and worst with different devices on different days.

## 3. Discussion

We compared OSA diagnostic data obtained in our clinical setting to oximetry data obtained in the Nagahama Study.

The slopes of the regression equations of the Bland–Altman plots (Figure 3) suggested similarities in the agreement of the clinical sleep tests, especially between PSG and type 3 OCST. The slope comparing oximetry and type 3 OCST (Figure 3b) was −0.794, which was between −0.809 (Figure 3a: oximetry vs. PSG) and −0.590 (Figure 3c: PSG vs. others). We did not directly compare PSG and OCST in the same patients in this study; however, the slope data suggested that increasing the number of monitoring channels from two or three in “Others” to four (as in the type 3 OCST) may provide more accurate data that is similar to PSG, the gold-standard test [9,26].

The differences in the correlation coefficients between our data (*r* = 0.561, *p* < 0.001) (Figure 2a) and Matsumoto et al. (*r* = 0.99, *p* < 0.001) [25] may be due to differences in settings. Our data compared previously performed oximetry with PSG or OCST that was performed later in a clinical setting. Matsumoto et al. compared ODI with AHI from simultaneously recorded data.

Previous studies compared AHI and ODI from the same recordings, with correlation coefficients reported to range between 0.745 and 0.97 [14,15,16,17,18,19]. Some studies compared simultaneously performed oximetry and PSG, with correlation coefficients reported to be 0.617–0.95 [20,21], while others independently performed oximetry and PSG [23,24], with correlation coefficients of 0.6 and 0.62. These results suggest that ODI compared with AHI from the same night may provide a better correlation. These trends in correlation coefficients were also found in our data; correlation coefficients between REI/AHI and ODI derived from the same recordings were 0.917 and 0.901, respectively; correlation coefficients between REI and ODI from different nights with the same device were 0.804 and 0.725; and the correlation coefficient between AHI/REI and ODI from different nights with different devices was 0.561.

Our results suggest that diagnostic performance of ODI to distinguish severe OSA from moderate OSA was not as effective as previously reported. The AUC to detect moderate-to-severe OSA (AUC = 0.736) was slightly higher than the AUC to detect severe OSA (AUC = 0.708) (Figure 4). Optimal thresholds to distinguish between moderate-to-severe OSA (ODI > 20.1) and severe OSA (ODI > 20.1) were the same, suggesting that use of ODI to distinguish between moderate-to-severe and severe OSA is difficult. Previous studies have suggested that patients with OSA detected by oximetry have more severe disease than those missed by it [14,23,27]. While these results suggest that oximetry is effective at diagnosing severe OSA, non-severe patients, including those with moderate disease, need PSG examinations for confirmation of their OSA [14].

OSA screening with oximetry is reported to have better performance than screening by questionnaire only [27]. Screening tests with oximetry may help participants to diagnose OSA with a shorter wait time than undergoing PSG in clinical settings [10].

Our study had some limitations. First, the oximetry ODI was not measured simultaneously with PSG/OCST. Comparisons of ODI and AHI/REI from the same recordings showed the best correlation [14,15,16,17,18,19] (Figure 5), followed by simultaneous recording by different devices [20,21,25]. The correlation was worst when comparing ODI and AHI/REI from different devices on different nights [23,24] (Figure 2a and Figure 6). This may be one reason why the correlation between ODI from previously performed oximetry and AHI/REI in our clinical setting was low (Figure 2a). The time period between oximetry in the Nagahama Study and PSG/OCST in our clinical setting was not clear because the participants only had ODI results and did not remember the exact day of their oximetry monitoring. This study was also performed in a single city in Japan, and the results may not be true for other populations. Moreover, only 11.4% of suspected OSA participants from the original study presented to our outpatient unit. However, it is reported that 11.0 to 16.5% of patients who underwent medical check-ups in Shiga, Japan, consulted referral doctors when recommended based on their results [28]. Thus, our participation rate may be reasonable in this regard.

## 4. Materials and Methods

### 4.1. Participants

Among the participants of the Nagahama Study, 12.1% (*n* = 933) had an ODI of ≥ 15 and were advised to undertake clinical sleep tests [25]. From October 24, 2013 to March 19, 2020, 129 participants visited our outpatient unit located at a tertiary care center in the Nagahama City Hospital for evaluation of OSA. Ten patients without ODI data were excluded, leaving 119 patients in our study. One hundred and six patients were reported to have an ODI ≥ 15, representing 11.4% of the Nagahama Study participants with an ODI of ≥ 15. The protocol of this study was approved by the Ethics Committees of the Nagahama City Hospital (27–37) and the Shiga University of Medical Science (R2015-229). This study was conducted per the Declaration of Helsinki in 2013. Written informed consent was not obtained because of the retrospective nature of the study. We disclosed the study protocol on the website (http://www.shiga-med.ac.jp/~hqsuimin/1207.pdf), and subjects were offered the opportunity to opt out of the study.

### 4.2. Sleep Tests and Questionnaires

Seven participants were not tested with PSG or OCST because they were not suspected to have sleep apnea, and two other patients refused PSG or OCST. One hundred and ten participants underwent PSG/OCST, and related data were further analyzed (Figure 1).

Twenty-two participants underwent in-lab PSG (Alice 5, Philips Respironics, Inc., PA, USA), 64 underwent type 3 OCST (Morpheus, Teijin, Tokyo, Japan), and 22 underwent 2-3 channel OCST (“Others”) (Figure 1). The type 3 OCST device monitored at least four channels and was defined as technically adequate to diagnose moderate-to-severe OSA [9]. We followed the recommended American Academy of Sleep Medicine (version 2.3) scoring criteria [29,30]. Apnea was defined as the cessation of airflow for at least 10 s, while hypopnea was defined as a reduction in the airflow amplitude or respiratory effort by at least 30%, with an oxygen desaturation value of 3% or greater for at least 10 s. AHI data were derived from the PSG test, while the respiratory event index (REI) was derived from OCST. REI is the term used to represent the frequency of apneas and hypopneas as measured by OCST. For OCST, we generally asked patients to record for two nights due to possible recording issues, a useful strategy for reducing the failure rate of OCST [1]. We had two OCST recordings for most participants, with an interval of 1.40 ± 0.877 days, and we were able to compare ODI and REI results from different nights for these same participants. Results for Figure 2, Figure 3, Figure 4 and Figure 5 were generated from recordings taken on the first night, while results for Figure 6 incorporated and compared data from both nights. The Japanese versions of the Epworth Sleepiness Scale (ESS) [31,32], Athens Insomnia Scale (AIS) [33,34], and Patient Health Questionnaire (PHQ)-9 [35,36] were used to assess sleepiness, insomnia, and depression, respectively. The ESS has eight items, and patients with an ESS of > 10 were classified as having daytime sleepiness. The AIS has eight items, and patients with an AIS of ≥ 6 or ≥ 10 were classified as having suspected or definite insomnia [37], respectively. The PHQ-9 is a reliable and validated instrument for screening individuals for major depressive disorders. In previous studies, participants with a PHQ-9 ≥ 10 were classified as having depression [35,36].

### 4.3. Statistical Analysis

Descriptive statistics for clinical characteristics were presented as mean ± standard deviation. Continuous and categorical variables were evaluated with an unpaired t-test or a chi-square test, respectively. Scatter plots and Bland–Altman plots were generated to compare ODI and AHI or REI. Receiver operating characteristic (ROC) curves were constructed to compare the diagnostic performance of ODI in detecting moderate-to-severe (AHI/REI ≥ 15) or severe (AHI/REI ≥ 30) OSA. Cohen’s kappa was used to analyze the agreement between OSA severities determined by either oximetry or PSG/OCST. A kappa value of 0–0.2 was considered to have essentially no agreement, 0.2–0.4 low agreement, 0.4–0.6 moderate agreement, 0.6–0.8 high agreement, and 0.8–1.0 nearly perfect agreement. Statistical analyses were performed using MedCalc version 19.2.1 (MedCalc Software, Mariakerke, Belgium).

## 5. Conclusions

Oximetry may be a useful tool for screening patients for moderate-to-severe or severe sleep apnea in large epidemiological studies. However, it may be difficult to set an appropriate threshold to distinguish moderate from severe sleep apnea using oximetry alone.

## Figures and Tables

**Table 1:** Patient characteristics. Characteristics of included and excluded participants were compared.

	Included	Excluded	*p*-Value
n	110	19	
Age, y	67.6 ± 10.3	69.6 ± 6.00	0.413
Sex, male %	69.1	63.2	0.609
BMI^1^, kg/m^2^	24.6 ± 4.07	24.7 ± 3.04	0.908
ESS^2^	6.43 ± 4.41	5.68 ± 3.07	0.482
AIS^3^	3.64 ± 3.15	3.58 ± 2.57	0.940
PHQ-9^4^	2.34 ± 3.18	1.92 ± 1.85	0.648
REI^5^/AHI^6^, per h	28.1 ± 15.0	28.1 ± 17.4	0.999
Hypertension, %	53.6	57.9	0.732
Diabetes, %	16.4	15.8	0.740
Dyslipidemia, %	33.6	31.6	0.861
Mental disorders, %	6.6	0	0.343

^1^ BMI: body mass index; ^2^ ESS: Epworth sleepiness scale; ^3^ AIS: Athens insomnia scale; ^4^ PHQ-9, Patient Health Questionnaire-9; ^5^ REI: respiratory event index; ^6^ AHI: apnea-hypopnea index.

**Table 2:** Sleep apnea severity distribution from oxygen desaturation indices (ODIs) obtained in the Nagahama Study and apnea-hypopnea indices (AHIs)/respiratory event indices (REIs) obtained in the clinical setting.

		Oximetry ODI					
		0–5	5–15	15–30	≥30	Total	%
PSG^1^/^2^OCST^2^ AHI/REI	0–5	0	2	1	0	3	2.7
	5–15	0	3	14	1	18	16.4
	15–30	0	2	42	1	45	40.9
	≥30	0	2	31	11	44	40
	total	0	9	88	13	110	
	%	0	8.2	80	11.8		

^1^ PSG: polysomnography; ^2^ OCST: out-of-center sleep testing.

## Abbreviations

AHI	Apnea-hypopnea index
AIS	Athens insomnia scale
AUC	Area under the ROC curve
ESS	Epworth sleepiness scale
OCST	Out-of-center sleep testing
ODI	Oxygen desaturation index
OSA	Obstructive sleep apnea
PHQ	Patient health questionnaire
PSG	Polysomnography
REI	Respiratory event index
ROC	Receiver operating characteristics
